# A Comparative Review of Eugenol and Citral Anticandidal Mechanisms: Partners in Crimes Against Fungi

**DOI:** 10.3390/molecules29235536

**Published:** 2024-11-23

**Authors:** Zinnat Shahina, Tanya E. S. Dahms

**Affiliations:** Department of Chemistry and Biochemistry, University of Regina, 3737 Wascana Parkway, Regina, SK S4S 0A2, Canada

**Keywords:** essential oils, eugenol, citral, *Candida albicans*, anticandidal mechanism, combinatorial action

## Abstract

*Candida albicans* is an emerging multidrug-resistant opportunistic pathogen that causes candidiasis, superficial infections on the mucosa, nails or skin, and life-threatening candidemia in deep tissue when disseminated through the bloodstream. Recently, there has been a sharp rise in resistant strains, posing a considerable clinical challenge for the treatment of candidiasis. There has been a resurged interest in the pharmacological properties of essential oils and their active components, for example, monoterpenes with alcohol (-OH) and aldehyde (-CHO) groups. Eugenol and citral have shown promising in vitro and in vivo activity against *Candida* species. Although there is substantial research on the efficacy of these essential oil components against *C. albicans*, a detailed knowledge of their mycological mechanisms is lacking. To explore the broad-spectrum effects of EOs, it is more meaningful and rational to study the whole essential oil, along with some of its major components. This review provides a comprehensive overview of eugenol and citral anticandidal and antivirulence activity, alone and together, along with the associated mechanisms and limitations of our current knowledge.

## 1. Introduction

The major opportunistic fungal pathogen *Candida albicans* typically resides on and within the human host as a commensal organism, but overgrowth of the natural host niche can lead to debilitating mucosal infections and systemic bloodborne infections associated with significant mortality [1]. Candidiasis is most frequent in patients who are immunocompromised or have medically implanted devices [2,3]. In recent years, anticandidal resistance has become a concern worldwide. Antifungal resistance provides a competitive advantage, arising from mutations in response to selection pressure, most of which involve inhibition of proteins or overexpression of efflux pumps that are responsible for shuttling antifungals out of cells [4,5]. The morphological plasticity of *C. albicans*, including hyphal and biofilm formation, makes it far more resistant to antifungal agents and difficult for the host immune system to mount an appropriate response [6]. Moreover, biofilm persister cells contribute to antifungal resistance through their gene expression, chromosomal abnormalities, overexpression of multidrug efflux pumps and increased membrane sterols, all contributing to antifungal tolerance and resistance [7,8].

With an alarming rise in the incidence of anticandidal drug resistance, it has become a serious challenge to develop novel drugs and discover new targets for this opportunistic fungal pathogen [9]. Hence, it is vital to explore alternative antifungal agents that are potentially active against *C. albicans*. The idea of using less expensive natural compounds having fewer side effects and often generating less resistance as antifungals has gained momentum [10]. Essential oils (EOs), volatile oily liquids extracted from the leaf, twig, roots, wood pulp or bark tissue of medicinal plants, are an excellent source of bioactive compounds. As secondary metabolites of plants, each EO is a chemically complex mixture consisting of more than 20–60 components of varying concentration, including terpenes, terpenoids (isoprenoids), aromatic and aliphatic aldehydes and phenols [11]. EOs are widely used for their bactericidal, fungicidal, antiviral, antiparasitic, insecticidal, and medicinal properties and cosmetic applications [12]. These low-molecular-weight compounds are highly lipophilic, which facilitates their entry into the cell membrane, leading to alterations of the cell architecture, the leakage of cell contents, and potentially cell death [13]. In the majority of cases, EO bioactivities closely relate to the activity of the predominant EO components [14]. Thus, it is more meaningful to study the whole essential oil, along with some of its major components. Currently a vast majority of research in the field has focused on the main oil components and their associated mechanisms. Among these components, eugenol and citral have been widely tested on microbes, including fungi, and their antifungal synergy underscores the need to understand their respective mechanisms. This review consolidates recent advances in our understanding of the anticandidal mechanisms of eugenol and citral and summarizes the latest approaches used to elucidate antifungal mechanisms.

## 2. Sources of Eugenol and Citral

**Figure 1:**
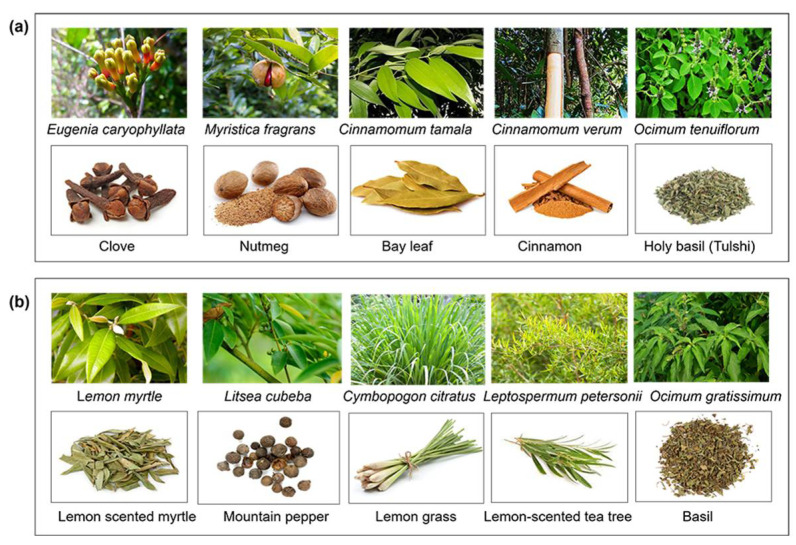
Natural sources of eugenol (**a**) and citral (**b**). The upper images indicate their scientific names and the lower images are common names.

Eugenol is found in a variety of plants (Figure 1), including *Eugenia caryophyllata* (clove), *Myristica fragrans* (nutmeg), *Cinnamomum tamala* (Indian bay leaf), *Cinnamomum verum* (cinnamon), *Ocimum basilicum* (common basil), *Ocimum tenuiflorum* (holy basil/tulsi), *Lippia multiflora* (lippia tea), *Mentha piperita* (peppermint), *Curcuma longa* (turmeric), and *Zingiber officinale* (ginger) [15,16]. Clove (*Eugenia caryophyllata* syn *Syzygium aromaticum*), consisting of eugenol (70–85%), eugenol acetate (15%), and caryophyllene (5–12%), is considered a major source of eugenol [17,18]. This volatile compound has a wide spectrum of biological properties used in personal care products, such as a local antiseptic and anesthetic, and as an antimicrobial in the food industry [15].

Citral is another key component of essential oils found in many plants (Figure 1), including *Lemon myrtle* (90–98%), *Litsea citrata* (90%), *Litsea cubeba* (70–85%), *Cymbopogon citratus* (65–85%), *Leptospermum petersonii* (70–80%), *Ocimum gratissimum* (66.5%), *Lindera citriodora* (~65%), *Calypranthes parriculata* (~62%), petitgrain (36%), *lemon verbena* (30–35%), *Eucalyptus staigeriana* (26%), and *Melissa officinalis* (11%) [19]. Citral is one of the most common flavoring compounds widely used in food production and as a raw material in the pharmaceutical, perfume, and cosmetics industries [20].

## 3. Chemical Structure and Characteristics of Eugenol and Citral

**Figure 2:**
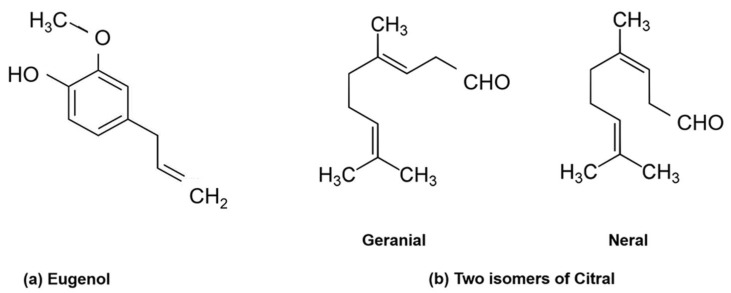
Chemical structures of eugenol and citral.

The phenylpropanoid eugenol (4-Allyl-2-methoxyphenol, Figure 2a) is a phenol that is partially soluble in water and its solubility increases with organic solvents, whereas the monoterpene citral ((2*E*)-3,7-dimethylocta-2,6-dienal, Figure 2b) is the name given to a mixture of two isomers: 40–62% geranial (trans-citral, citral A) and 25–38% neral (cis-citral, citral B), acyclic α,β-unsaturated monoterpene aldehydes [21]. Citral is insoluble in water, but soluble in ethanol, mineral oil, and diethyl ether [22].

### 3.1. Antimicrobial Activity of Eugenol and Citral

Eugenol has a wide range of activity [15,23,24,25,26] against bacteria (*Staphylococcus aureus*, *Pseudomonas aeruginosa*, *Enterobacter aerogenes*, *Listeria monocytogenes*, *Escherichia coli*, *Streptococcus agalactiae*, *Klebsiella pneumoniae*), fungi (*Candida* spp., *Aspergillus niger*, *Penicillum glabrum*, *P. italicum*, *Fusarium oxysporum*, *Saccharomyces cerevisiae*, *Trichophyton mentagrophytes*, *Lenzites betulina*, *Laetiporus sulphurous*, and *Trichophyton rubrum*.), viruses (Herpes simplex virus 1 and 2) and protozoa (*Leishmania donovani*, *Plasmodium falciparum*, *Trypanosoma cruzi*, *Giardia lamblia*, *Schistosomes* spp.). The antimicrobial activity of eugenol is ascribed to the hydrophobic aromatic ring and free hydroxyl group [27,28], which modify the fluidity and permeability of cell membranes, leading to leakage of cytoplasmic contents [24]. Eugenol also reduces ergosterol content, interferes with H^+^-ATPase activity, induces oxidative stress [24], and interferes with cell adhesion, as well as biofilm formation and development [23].

An appreciable number of studies show that citral can inhibit the growth of Gram-positive and Gram-negative bacteria as well as fungi, including *Staphylococcus aureus*, *Escherichia coli*, *Cronobacter sakazakii*, *Salmonella typhimurium*, *Listeria monocytogenes*, *Aspergillus niger*, *Nannizzia gypseum*, *Trichophyton mentagrophytes*, *P. italicum*, *P. digitatum* and *Candida* spp. [29]. The proposed antimicrobial mechanism of citral includes cell membrane dysfunction, inhibition of respiratory enzymes, dissipation of the proton-motive force [30], leakage of cellular constituents [31,32] and finally cell death. Like eugenol, citral exhibits potent activity against microbial biofilms [33], but this antivirulence mechanism has not been well characterized.

### 3.2. Anticandidal Mechanism of Eugenol and Citral

*C. albicans* responds to eugenol and citral with some common mechanisms and several that are different.

### 3.3. Impact on the Candidal Cell Wall

The *C. albicans* cell wall, which facilitates adhesion, colonization, cell morphology, integrity and viability [34], makes an excellent antifungal target based on its unique antigenic components. External cell wall proteins [35] attach to an inner polysaccharide layer with 60% glucan (β-1,6-glucan (20%), β-1,3-glucan (40%)), 1–2% chitin and other minor sugars [36]. Chitin biosynthetic inhibition has negative impacts on cell wall maturation, septum formation, and bud ring formation and can lead to lysis and cell death [37].

Eugenol significantly increases chitin content in *C. albicans*, which is typically a response of the cell wall integrity pathway, a general stress response [38]. At higher concentrations, changes to hydroscopic turgor pressure inside the cells [39] cause their deflation [40], which can be detected by scanning electron microscopy (SEM) and atomic force microscopy (AFM). Conversely, citral does not act on the cell wall of *C. albicans* or any other fungal spp. [32,41].

### 3.4. Cell Membrane Permeabilization and Dysfunction

The *C. albicans* cell membrane, which maintains osmotic support for turgor pressure [42], consists of a diverse set of lipids including phospholipids, glycerophospholipids and sterols [43]. Ergosterol, the main sterol component of the candidal cell membrane [44], coordinates membrane fluidity, permeability and integrity in *Candida* [45]. The cell membrane also houses major protein and enzyme families involved in stress responses, transport, signal transduction, carbohydrate metabolism and cell wall biosynthesis [46]. Moreover, the cell membrane plays critical roles in the secretion of virulence factors, endocytosis and invasive hyphal morphogenesis [46,47]. Therefore, small molecules that target ergosterol synthesis and damage membrane permeability are suitable antifungals [48].

Both eugenol and citral at higher concentrations (1000 and 256 µg/mL, respectively) make the *C. albicans* cell membrane permeable in a dose-dependent manner, ultimately leading to cell death [38], consistent with reports linking cell membrane damage [32,49] to reduced membrane potentials and disruption of proton pumps [39]. Many lipid-soluble EO components, including eugenol, inhibit ergosterol biosynthesis, interfering with membrane integrity and functionality [50,51,52,53,54]; however, only citral can inhibit ergosterol biosynthesis in *Candida* spp. [41].

### 3.5. Inhibition of Efflux Pumps

Fungal efflux is crucial for the removal of harmful compounds (e.g., antifungals) from the intracellular environment, and efflux pumps support the transmembrane electrochemical proton gradient that is necessary for nutrient uptake [55]. Thus, efflux systems are critically important in anticandidal resistance for both planktonic forms and those within biofilms [56,57]. Eugenol significantly inhibits H^+^-ATPase activity and glucose-stimulated H^+^ extrusion, causing intracellular acidification and cell death in *C. albicans* [58]. The eugenol derivative, eugenol tosylate, directly binds to the efflux pump and inhibits H^+^-ATPase activity to various degrees [59], and so can mitigate fluconazole resistance resulting from efflux. On the other hand, there is no evidence for citral in this regard.

### 3.6. Mitochondrial Dysfunction

Mitochondria, specialized organelles found in all eukaryotes, contribute to energy production through the electron transport chain and therefore produce reactive oxygen species (ROS) [60], but also maintain cell physiology by controlling the cell cycle, cell growth, signaling, ion homeostasis and metabolite production [61]. Consequentially mitochondria influence the yeast-to-hyphal morphological transition, cell wall biogenesis, virulence, and antifungal resistance [62], and are therefore a target for antifungal development [63,64]. Both eugenol and citral generate mitochondrial dysfunction by inhibiting NADPH biosynthesis and other mitochondrial enzymes in *Aspergillus flavus*, *Trichophyton mentagrophytes*, *Tagetes patula* and *P. digitatum* [65,66,67]. At high concentrations, eugenol and citral disrupt *C. albicans’s* mitochondrial membrane potential, leading to increased ROS levels and microtubule catastrophe [38], linking mitochondrial ergosterol depletion [68] and hyperpolarization [69].

### 3.7. ROS Induction

Mitochondria are the origin of ROS, a normal physiological condition in eukaryotes [70], but also play important roles in cellular apoptosis [71], essential for the development and survival of unicellular and multicellular eukaryotes [72,73] by mitigating metabolic disorders [74]. Eugenol and citral at higher concentrations both cause ROS-dependent cell death by disrupting the membranes of vacuoles, mitochondria and nuclei, resulting in microtubule disruption and cell cycle arrest [38,75,76]. While a short exposure (4 h) time is sufficient for eugenol to induce ROS elevation, citral requires a longer time (24 h) to cause oxidative stress in *C. albicans*.

### 3.8. Vacuolar Membrane Damage

Vacuoles play multiple roles in protein and membrane trafficking, osmoregulation, cell volume regulation, stress response, and intracellular ion homeostasis, all important for adaptation to new environments and survival under stressful conditions [77]. Notably, inheritance defects, with an acute block in vacuole biogenesis and loss of function, result in cell cycle arrest [78]. Moreover, in *C. albicans*, vacuolar expansion and germ tube formation strategically aid in rapid hyphal morphogenesis required for virulence [79]. Disruption of host membrane integrity and therefore the storage and regulation of hydrolytic enzymes by the V-ATPase vacuole protein sorting 11 (Vps11) subunit [79,80] highlights how ion homeostasis is an effective antifungal target. Strikingly, higher doses of eugenol and citral disrupt vacuolar membrane integrity, which induces cell cycle arrest and loss of cell viability, and is consistent with the accumulation of clove oil (a major source of eugenol) in *C. albicans* vacuoles, cell wall, membrane, and cytoplasm [81]. However, at sublethal levels, citral and eugenol also disrupt vacuole, hyphal, and biofilm formation, but in an ROS-independent manner [38].

### 3.9. Cell Cycle Arrest

The cell cycle is associated with a series of events that tightly regulate the generation of new daughter cells through the duplication and partition of genetic information and cellular components, consequently helping to regulate and maintain hyphal morphogenesis in *C. albicans* [82,83]. The progression is triggered by the activation of cyclin proteins, and is controlled by the cell wall integrity pathway [84,85]. Environmental signals or stress (e.g., antifungal drugs, DNA damage) can trigger polarized growth as a result of cell cycle arrest in *C. albicans* [86,87], with vacuole impairment causing arrest specifically at the early G1 phase [78]. Both citral and eugenol can cause cell cycle arrest at the S or G1 phase in *C. albicans* [38,49,88], confirming the link between membrane stress and vacuolar defects [38].

### 3.10. Microtubular Disruption

Microtubule (MT) networks of *C. albicans* consist of α- and β-tubulin heterodimers responsible for a variety of functions, including sustaining cell shape, intracellular transport, cell division [89] and the regulation of hyphal growth [90]. Of note, defects in MT morphology relate to disruption of hyphal elongation, with the nuclear cell cycle coordinated by hyphal length or volume [91]. The MT apparatus has been identified as a viable antifungal target [92], and, interestingly, both eugenol and citral delocalize Kar3p, a member of the kinesin-14 family critical for MT stabilization and hyphal and biofilm formation [93]. Kar3p is required for normal mitotic division and morphogenesis in *C. albicans* and its delocalization is associated with irregular mitotic spindles, leading to prolonged cell cycle arrest known to induce pseudohyphal development [94]. MT disorganization by eugenol has been directly linked to ROS [95]; however, citral does not elevate ROS, implying a direct impact on MT formation. Indeed, MT disruption is an important factor for hyphal impairment, supported by microscopy and computational modeling showing the theoretical binding of citral and eugenol adjacent to the cofactor-binding sites of αβ-tubulin and Kar3p [93]. Despite many unanswered fundamental questions in relation to MT defects, MT disruption appears to be a promising new avenue for anticandidal design.

### 3.11. Hyphae and Biofilm Inhibition

Germ tube formation, cell adhesion and subsequent hyphal and biofilm formation, respectively, are all *C. albicans* attributes proposed to govern pathogenicity [96], and are important factors in severe antifungal resistance [97]. Thus, targeting these processes and their associated machinery are promising avenues for overcoming drug resistance or preventing host invasion through medical devices.

The cell cycle not only plays a major role in regulating cellular morphogenesis and vacuolar biogenesis, but is an important factor for hyphal morphogenesis at the G1 phase [78,98]. Hyphal development requires the complex movement of nuclei through the bud neck during the initial stages of the cell cycle, orchestrated by MTs [90,91], and any interruptions by antifungal drugs can arrest cell proliferation [99,100]. Biofilms, as communities of adherent organisms with a complex three-dimensional structure enshrouded by extracellular matrix, are key contributors to candidal pathogenesis regulated by specific genetic programs and metabolic processes [96], offering up to 1000-fold more resistance to antifungals compared to planktonic cells [101,102].

Therefore, targeting hyphal and biofilm formation as opposed to cell viability may be particularly appealing for combating candidal infections and reducing resistance. Eugenol inhibits the formation of hyphae and mature biofilm in drug-resistant *C. albicans* [103,104,105,106] and interferes with adhesion and invasive growth [88]. Similarly, citral has potent activity against *Candida* mycelial, hyphal and biofilm growth [104,107,108,109,110]. A link has been established between ROS-independent Kar3p delocalization, MT defects and hyphal inhibition for *Candida* treated with citral and eugenol at sublethal concentrations [38]. Furthermore, citral and eugenol both impair mitochondrial activity, leading to repression of *C. albicans* filamentation and biofilm development, supporting the idea of chemically induced inhibition of morphological transitions [111,112]. Earlier work highlights the link between mitochondrial respiration and biofilm development [113,114] using strains lacking mitochondrial NADH dehydrogenase.

The disruption of cell morphology and biofilm by citral and eugenol may relate to specific proteins responsible for biofilm or hyphal formation.

Many cell wall proteins (e.g., Als1, Als2, Als3, Hwp1) along with their transcriptional activators and repressors (*EFG1*, *TEC1*, *BCR1*, *TUP1* and *NRG1*) [115] facilitate biofilm formation, cell–cell aggregation and host cell invasion [116], thus impacting primary *C. albicans*–host interactions and pathogenesis. Thus, EO-induced cell membrane defects could prevent cell wall proteins from reaching their destination at the cell wall, and this idea is supported by computational studies revealing their theoretical interactions [117], in particular Als3, an adhesin ultimately responsible for biofilm formation.

### 3.12. Towards a Mechanistic Understanding of Anticandidal and Antivirulence Effects of Eugenol and Citral

**Figure 3:**
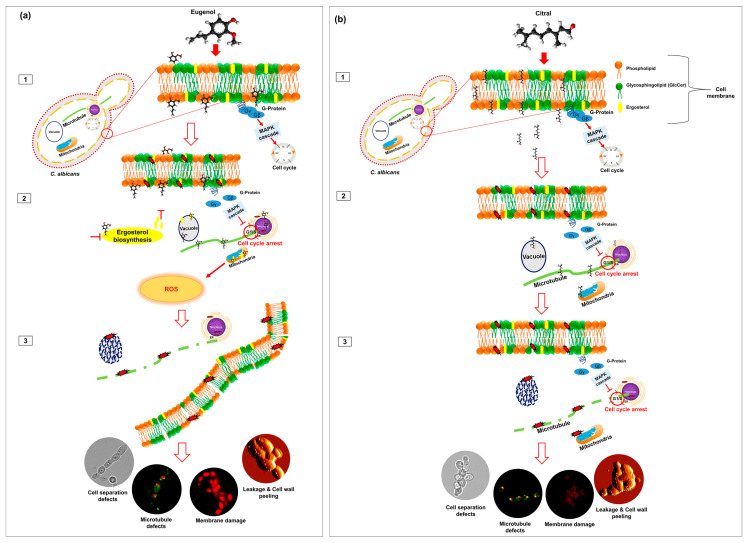
Proposed model for the anticandidal activity of eugenol and citral after short-term exposure. (**a**) We propose the anticandidal mechanism of eugenol as a simplified model showing (1) eugenol entering the cell membrane, causing membrane depolarization and ergosterol binding, with membrane defects leading to immediate cell cycle arrest at the G1/S phase. In (2), eugenol’s phenolic group facilitates its entry into the cytoplasm, disrupting ergosterol biosynthesis and protein trafficking, which triggers mitochondrial imbalance and the overproduction of ROS. (3) Loss of mitochondrial membrane function causes accumulation of intracellular ROS, which can damage nucleic acids, MTs, and the cell membrane and cause leakage of intracellular contents and cell death. (**b**) The proposed anticandidal mechanism of citral shows it (1) easily penetrating the cell membrane, causing membrane depolarization and immediate cell cycle arrest at the G1/S phase. (2) Once the cell membrane is saturated, citral passes into the cytosol where it preferentially partitions into MTs and vacuolar and mitochondrial membranes. (3) Loss of organelle membrane potential causes vacuolar segregation and disrupts the mitochondrial proton gradient, hampering the normal function of these organelles. On the other hand, association with MTs causes defects in cell separation and induces pseudohyphal growth.

Despite eugenol and citral having similar anticandidal mechanisms, the chemical structure of EO components can influence their absorption, biological activity and metabolism [118]. EO components can be categorized by their functional groups and carbon skeleton (cyclic versus aliphatic). For example, eugenol’s aromatic ring enables its association with the lipid bilayer fatty acyl chains, disrupting membrane potential, fluidity and permeability [119], while its phenol substituent (-OH) enables hydrogen bonding that can perturb the membrane and associated enzymes [27,120,121]. On the contrary, the aliphatic aldehyde citral has been proposed to act solely by permeabilizing the membrane [122,123], despite its ability to form charge transfer complexes with tryptophan [124,125]. Several studies suggest that eugenol not only binds ergosterol (Figure 3a) but inhibits its synthesis [126,127]. The aromatic ring structure of eugenol enables its accumulation in the membrane, which may allow it to bind ergosterol. On the other hand, citral inhibits ergosterol biosynthesis in *Candida* spp. [41] but without directly binding ergosterol. Thus, it is apparent that citral can easily penetrate and pass through the cell membrane (Figure 3b) with a minimum dose, whereas the aromatic ring of eugenol helps to trap it in the membrane, requiring a larger dose to gain entry into the cell.

Eugenol and citral both initiate apoptosis by the same mechanism. With short exposures at high concentrations, both are capable of saturating, depolarizing, and passing through the cell membrane to access organelle membranes, hampering ergosterol biosynthesis and reducing the activity of H^+^-ATPase and cytosolic proteins. However, for citral, unlike eugenol, this is an ROS-independent process, and it is difficult to understand why eugenol generates elevated ROS but citral does not. It is reasonable to propose that citral can immediately arrest the cell cycle at the G1/S phase by more quickly passing through the cell membrane and interfering with MTs and vacuolar and mitochondrial membranes, causing candidal dysfunction at various levels (Figure 3b). On the other hand, eugenol, which is proposed to directly bind ergosterol, significantly alters cell and organelle membrane fluidity, potentially inhibiting protein trafficking and sorting to the cell surface and triggering mitochondrial imbalance, causing ROS overproduction after a short exposure. In the cytosol, excessive ROS weakens the defense system of the mitochondrial membrane, which subsequently elevates the cellular oxidant potency to further contribute to ROS and oxidative bursts, leading to further damage of essential cellular components including the vacuoles, nucleus, and MTs (Figure 3a), and ultimately resulting in cell death. However, inhibition of hyphal and biofilm formation with sublethal levels of EO components is a completely ROS-independent mechanism, with subsequent impairment of vacuoles, mitochondria, and MTs.

### 3.13. Combinatorial Effects of Eugenol and Citral

Combination therapy is proposed to avoid or mitigate anticandidal resistance, while reducing the required dose of each, which in turn reduces side effects [128].

While synergism is optimal, partially synergistic or additive combinations still serve to lower the minimal required dose, and thus can also be good choices for combination therapy [129].

The combinatorial impact of conventional antifungal agents with eugenol or citral is well documented [105,110,130,131,132,133,134], but the antivirulence capacity of essential oils, and in particular their constituents in combination, against *C. albicans* remains largely unexplored. Citral and eugenol have different anticandidal mechanisms and therefore should have greater efficacy in combination. Together, they are synergistic against *Shigella flexneri*, *Aspergillus niger*, and *Penicillium roqueforti* [135,136,137] and additive against *C. albicans* [38]. Eugenol and citral can enhance the effects of other antifungal agents; for example, a combination of eugenol or citral (120–150 and128 µg/mL, respectively) with 0.31–0.53 or 1 µg/mL of fluconazole, respectively, was effective against most fluconazole-resistant *Candida* isolates. This effect is assumed to be the sensitizing nature of eugenol and citral, which allows fluconazole to penetrate the fungal cells more effectively and inhibit biofilm formation [110,130].

### 3.14. Limitations and Future Strategies for EO Components as Antifungals

Although eugenol and citral have strong antimicrobial activity, many clinical reports indicate nausea, dizziness, convulsions, rapid heartbeat [138,139], irritation and sensitization of human skin [140]. Substantial research has reported the anticancer activity of essential oils against breast, hepatoma, colon, and submandibular cancer and melanoma cells [138,141,142,143] through cell membrane damage, free radical generation, reduced energy metabolism, apoptosis, cell cycle arrest and loss of key organelle function [144,145]. A recent study demonstrates that EO components are not only toxic to cancerous cells but also non-cancerous fibroblast cells [145] that produce and maintain the extracellular matrix in connective tissues and are present in every tissue of the body except the blood [146]. Therefore, the entire body would be affected if EO components were used for cancer treatment, unless in a directed fashion, or invasive fungal infections. In fact, citral concentrations higher than 1% are cytotoxic to human fibroblast cells [147]. Nevertheless, cytotoxicity may be mitigated at sublethal concentrations capable of inhibiting hyphal and biofilm formation.

Although the majority of EOs are not carcinogenic, some of their components can be secondary carcinogens [148,149] resulting from oxidative stress [150]. Thus, toxicological studies are key when considering formulations of EO components to prevent *Candida* infection. One challenge in interpreting the literature on antimicrobials is the variation in interventions across studies. For example, doses and methods are not uniform and very few randomized control trials exist for the evaluation of their comparative efficacy. Recent advances in genomic and proteomic methods, such as comparative gene expression, microarray, and comparative proteomic and genomic methodologies, could elucidate and explain some key missing pieces.

## 4. Conclusions

Essential oils have been widely used for their calming, stimulating, anti-inflammatory, analgesic, anti-bacterial, fungicidal, medicinal and cosmetic properties by the pharmaceutical, sanitary, cosmetic, food, and agricultural industries for decades. The complex blends of compounds within each essential oil have made them holistic aromatherapies since antiquity, but they are now well established for the treatment of various infections and conditions. Although eugenol and citral share similar anticandidal mechanisms, the phenolic group makes eugenol more potent than citral. Both are potentially promising bioactive compounds, with broad-spectrum anticandidal activity against planktonic and sessile cells; however, there is insufficient evidence of treatment effects and effectiveness in humans under most of the conditions studied. Nevertheless, the ability to inhibit hyphal and biofilm formation at sublethal concentrations holds promise, and offers a foundation for future studies examining synergism between classical antifungals and EO components. Developing novel EO-based sanitizing formulations and medical coatings is a promising avenue for reducing infection rates and prophylaxis.

## Data Availability

No new data were created or analyzed in this study. Data sharing is not applicable to this article.
